# Assessing the Moderation Effects of Socioeconomic Factors in Predicting Severe Crash Outcomes Based on Road and Environment Characteristics: A Modeling Comparison

**DOI:** 10.1177/03611981261423464

**Published:** 2026-03-24

**Authors:** Mahsa Jafari, Bhagwant Persaud

**Affiliations:** 1 Department of Civil Engineering, Toronto Metropolitan University, Toronto, Ontario, Canada

**Keywords:** crash severity, structural equation modeling, moderation, machine learning, Shapley additive explanation algorithm

## Abstract

Despite the increasing use of structural equation modeling (SEM) and machine learning (ML) techniques in traffic safety analysis, few studies have integrated these approaches to examine how socioeconomic factors moderate the relationships between crash severity and road/environmental characteristics. This study addresses that gap by applying a two-stage hybrid modeling framework that combines partial least squares structural equation modeling (PLS–SEM) with ML techniques to analyze the propensity of road segments to experience severe crashes, considering the moderating effects. A data set of lane departure crashes on Ohio collector roads and associated roadway, weather, and socioeconomic data was used as a case study. In the first stage, PLS–SEM was used to model latent constructs and investigate the moderation effects of socioeconomic variables. The second stage applied four ML methods: (1) random forest; (2) support vector machines; (3) artificial neural networks; and (4) eXtreme Gradient Boosting, to improve predictive performance and identify nonlinear relationships. The results indicate that the SEM–RF combination outperformed the others, achieving higher scores in macro average F1-score, precision and recall. Subsequent analysis with the Shapley additive explanation algorithm (SHAP) revealed that some moderators were among the top contributors to crash severity, insights not captured by SEM alone. By revealing how socioeconomic factors interact with roadway and environmental factors associated with severe crash outcomes in nonlinear ways, this hybrid approach enables tailored, data-driven safety interventions. The findings can support developing targeted countermeasures that reflect the unique socioeconomic conditions of each neighborhood, advancing more equitable and effective traffic safety strategies.

## Introduction

According to the World Health Organization, in 2021, there were approximately 1.19 million fatalities because of road traffic accidents, equal to a rate of 15 deaths per 100,000 individuals (*1*). These statistics highlight the urgent need to not only comprehensively identify and understand the factors that contribute to the severity of traffic crashes but also to continuously enhance the methods used to analyze and mitigate these factors.

The analysis can be complicated because crash outcomes are influenced by multiple interacting variables (*2*), including human characteristics, road conditions, and crash attributes (*3*). In a road environment, socioeconomic factors of different communities can also affect traffic crashes (*4* [Bibr bibr5-03611981261423464][Bibr bibr6-03611981261423464][Bibr bibr7-03611981261423464]–*8*). However, few studies have evaluated the effect of socioeconomic variables as third variables on the relationships between dependent and independent variables (i.e., moderation effects).

Undertaking methodological advances in traffic crash analysis can be challenging. This is largely because traditional statistical models are often not well-suited to analyze crashes because of some limitations, such as the inability to capture latent constructs, moderation effects, or to perform multigroup analyses. One approach for addressing these limitations is partial least squares structural equation modeling (PLS–SEM), a widely utilized statistical method for exploring complex relationships, while including latent variables and performing moderation and multigroup analysis (*9*, *10*).

Despite its advantages, SEM typically simplifies complex decision-making processes by establishing linear relationships; however, these relationships can be nonlinear and otherwise complicated (*11*). This issue can be addressed by applying machine learning (ML) methods, which can capture complex relationships between variables (*12*, *13*). It is, however, inappropriate to use ML techniques to test hypotheses because they are “black-box” approaches. Therefore, it is recommended (*14*) that a linear SEM model is complemented with a nonlinear ML model to balance the strengths and weaknesses of these two approaches and to offer a more comprehensive framework. SEM allows for the inclusion of latent variables and the modeling of both direct and indirect effects, as well as the evaluation of moderation without the rigid functional assumptions required in regression. In addition, ML techniques can complement SEM by capturing nonlinear relationships and complex interactions, enhancing predictive performance when linear assumptions do not hold. Combined, these methods address the shortcomings of traditional regression models, providing theoretical rigor and data-driven adaptability.

Previous studies have applied SEM or ML techniques independently to analyze crash severity; no study on traffic safety has combined these methods in a hybrid framework, to the best of the authors’ knowledge. Neither has any study conducted a comparative analysis of multiple ML models to identify the most effective approach when examining the moderating effects of socioeconomic factors. Therefore, the primary aim and central novelty of the research in this study was to advance this hybrid approach that combines the explanatory power of SEM with the capabilities of various ML methods in predicting crash severity outcomes. For this study, a database of Ohio collector road segments, details of which are provided in the data section, was used as an illustrative case study to assess the moderation effects of socioeconomic factors in predicting severe crash outcomes based on road and environmental characteristics. The exploration of these moderation effects adds to the novelty of this study, as does the comparative evaluation of various ML techniques used in conjunction with SEM.

Fundamentally, this study aims to answer the following questions with the use of the case study.

- Which ML method can perform better than others when applied in combination with SEM to predict whether road segments will have severe crash outcomes?
- What are the most important factors affecting the severity based on the best hybrid model?
- To what extent can the proposed hybrid SEM–ML framework reveal the moderating effects of socioeconomic variables on the relationships between crash severity and environmental/road factors?

To address these questions, the following steps are followed from a methodological aspect in this study.

1. The SEM model was developed to analyze linear moderation effects of socioeconomic factors on road environmental factors.
2. The hybrid SEM–ML techniques (including artificial neural networks [ANN], eXtreme Gradient Boosting [XGBoost], random forest [RF], and support vector machines [SVM]) were developed to detect nonlinear moderation effects. In addition, the model performance of these techniques was compared, and the best-performing model was identified.
3. Using the best-performing model, the feature importance analysis was performed using the Shapley additive explanation algorithm (SHAP) (*15*) to represent the nonlinear effect of several socioeconomic-roadway interactions.

## Literature Review

The literature review focuses on the application of SEM and ML techniques, including ANN, XGBoost, RF, and SVM, in traffic safety research and their hybrid applications. The Methodological Specifics section complement this review in providing more details on these techniques as they pertain to this study.

The SEM technique has been widely used in traffic safety for modeling complex relationships among latent and observed variables and testing moderation effects (*16* [Bibr bibr17-03611981261423464][Bibr bibr18-03611981261423464]–*19*). Moderation occurs when a third variable, known as the moderator, affects the strength or direction of the relationship between two constructs (*20*). For example, Haerani et al. (*18*) studied the moderating effect of age on the association between driving behavior, personality variables, and driving outcomes. Jing et al. (*19*) analyzed the effect of risk preferences, risk perceptions, and their interaction with risky driving behaviors and assessed the moderating effect of age, gender, and driving experience.

In the application of ML techniques, ANN (*2*, *13*), XGBoost (*21*, *22*), RF (*23*, *24*), and SVM (*25*, *26*) have been used extensively in traffic safety studies to effectively model complex relationships (*27*). There are several studies that compare the performance of these techniques. For example, Zhang et al. (*28*) compared the predictive performance of various ML and statistical models and found that ML methods are significantly more accurate than statistical methods but are susceptible to overfitting.

While ML methods handle complex relationships effectively, they struggle to accommodate latent variables or analyze moderating effects. In contrast, SEM is specifically designed to incorporate latent variables and analyze indirect effects. Therefore, hybrid approaches that combine SEM with ML techniques can offer more robust insights into complex relationships, including moderations between latent variables.

The hybrid applications of SEM have been increasingly adopted in different fields, such as business (*29* [Bibr bibr30-03611981261423464]–*31*), health (*32*), data analysis (*14*, *33*), and transportation (*34*, *35*). Of special relevance to this study is Jafari and Persaud (*35*), who proposed a two-stage hybrid SEM–ANN approach to jointly examine crash frequency and severity. While that study demonstrated how the combination of SEM and ANN could capture both latent and nonlinear relationships, it was limited to a single ML method (e.g., ANN). Therefore, comparing the predictive performance of several ML methods was still beyond the scope of that study. To address this limitation, this study aimed to apply a hybrid use of SEM and ML techniques to investigate the moderation effect of socioeconomic factors on the relationship between road and weather characteristics and crash severity. Therefore, this study was expected to explore this hybrid approach and to provide new insights into the complex relationships among various factors that are associated with crash severity outcomes.

## Data

The data set for the illustrative case study consists of 12,736 lane departure traffic crashes that occurred on 6,971 collector road segments in Ohio over 6 years. Saleem et al. (*8*) used these data to identify focus crashes and facility types, as well as contributing factors, to enhance the understanding and application of systemic safety improvements. Lane departure crashes were chosen as the focus of this study because they were identified as a focus crash type by Saleem et al. (*8*) in their application of the systemic safety approach. Collector road segments were chosen because they are designed to provide a balance between traffic mobility and land access, providing connectivity between arterials and other collectors (*36*); therefore, they are utilized by motorists who reside in adjacent neighborhoods that are characterized by socioeconomic factors that may affect driving behavior on these roads.

The data include information from three sources: (1) the Highway Safety Information System (HSIS) (*37*) for crash and roadway inventory data; (2) the National Oceanic and Atmospheric Administration (*38*) for weather data; and (3) the US Census Bureau (*39*) for socioeconomic census data.

The descriptive statistics of the database can be found in Table 1. Of note, more than half of the curves and grades fall under the categories of tangent and level, respectively. In addition, over half of the people in these communities earn more than $50,000/year and hold a higher education degree. Finally, crash severity serves as the dependent variable, representing whether a road segment has experienced a fatal or injury traffic crash. Therefore, crash severity is a binary variable, with segments having at least one crash designated as property damage only (PDO) or no apparent injury coded as zero, while 15% of segments experiencing at least one injury or fatal crash were coded as one. The variable descriptors are mainly taken directly from the original database and are self-explanatory.

**Table 1.** Descriptive Statistics and Variable Names

Variable description	Mean	SD	Minimum	Maximum	Variable name/category used in the analysis
Segment length (mi)	0.58	0.94	0.001	8.03	Ln(Segment Length)
Curve radius (ft)	69.6	195.6	0	1,311	radiusft_moderate (500–1,000 ft)
					radiusft_tangent (> 1,000 ft)
Grade percentage	2.22	3.78	0	20	grade steepness_level (0–3)
					grade steepness_steep (≥ 6)
Annual average daily traffic (AADT)	2,132	1,648	92	12,360	Ln(AADT)
Surface width (ft)	20.88	2.58	16	46	Surf_wid
Speed limit (mph)	52.78	5.33	25	60	speedlimit 40–50 mph
					speedlimit 50–60 mph
Total average rainfall and snowfall (in.)	69.7	21.73	48.2	158.64	average precipitation
Average annual maximum temperature (°F)	61.09	2.54	57	69	Tempmaxavg
Proportion of population ages 25+ years with a diploma and university degree	0.56	0.1	0.14	0.89	diploma and universityplus
Proportion of households with income > $50,000	0.51	0.14	0.03	0.96	income more than 50k
Proportion of households with at least one vehicle	0.94	0.09	0.12	1	vehicle 1 and more
Proportion of population ages 45+ years	0.46	0.1	0.04	0.83	Age45plus
Fatal and injury lane departure crashes	0.59	0.88	0	10	LNDP_KAB
No apparent injury and PDO lane departure crashes	2.7	2.7	0	45	na

*Note:* na = not applicable; PDO = property damage only; SD = standard deviation.

Of note, the socioeconomic indicators in the original data set were already provided as bracketed proportions, for example, the percentage of households in income ranges of $ 0–$50,000, $50–$100,000, and $100, 000 or more. However, the crash severity variable was intentionally defined as binary to align with the systemic safety framework (*8*). This approach allows the analysis to focus on identifying road segments that are more prone to severe crashes, consistent with proactive safety management practices. While this simplifies the analysis and improves interpretability, it also reduces some of the detail available in the raw data, which can be seen as a study limitation.

## Methodological Specifics

As noted, this study employed a two-stage approach in which SEM and ML methods are combined to investigate factors influencing traffic crash severity outcomes on road segments. In addition to evaluating linear moderation effects using SEM, the hybrid analytical framework incorporates ML models to detect nonlinear moderation patterns that are not identifiable using SEM alone. SHAP-based feature importance analysis is subsequently used to quantify the relative influence of these nonlinear interaction effects. This two-stage structure enables the combined SEM–ML approach to reveal moderation thoroughly. In the first stage, similar to Li et al. (*40*), hypotheses were tested, moderation effects were assessed, and latent scores were extracted for use in the second stage. During the second stage, various ML models were developed, their performances compared, and important factors, including moderators, affecting severity outcomes were ranked using the best model. The point of departure from Li et al. (*40*) is that all latent variable scores from the SEM, whether significant or not, were included as ML inputs, recognizing that non-significance in SEM may arise from nonlinear effects that ML algorithms can capture. The PLS–SEM and data mining models were developed using SmartPLS 4.1 (*41*) and Python, respectively.

### Structural Equation Model

PLS–SEM is a powerful method for analyzing complex relationships among latent variables. PLS–SEM was selected for this study because of its capability to analyze moderation and its suitability for handling skewed, non-normal data, conditions commonly present in crash and socioeconomic datasets. A structural model has two key components: (1) the measurement model (outer model); and (2) the structural model (inner model). The measurement model defines the relationships between latent variables (constructs) and their corresponding indicators (observed variables), while the structural model represents the relationships among the latent variables. The PLS–SEM algorithm begins by estimating initial latent variable scores, typically using ordinary least squares regression. It then calculates the outer model by determining the weights and loadings for the indicators associated with each latent variable. Subsequently, the inner model is estimated by computing the path coefficients that describe the relationships between latent variables, as well as the residuals for the dependent latent variables.

The PLS–SEM algorithm operates iteratively, refining the measurement and structural models. During each iteration, it updates the estimates of weights, path coefficients, and latent variable scores until convergence is achieved. This iterative process ensures that the model maximizes the explained variance in the dependent constructs. The measurement and structural models are presented in Equations 1 and 2 (*20*, *42*).

(1) $$X=\Lambda_{x}\times \;\xi +\epsilon_{x}$$

(2) η=Γ×ξ+ζ

where

*X* = vectors of observed variables for the constructs,

Λ_*x*_ = factor loadings for the constructs,

*ϵ*_*x*_ = measurement errors for the variables,

*η* = vector of dependent latent variables (dependent constructs),

*ξ* = vector of independent latent variables (independent constructs),

Γ = path coefficient matrix representing the relationships between latent variables, and

*ζ* = vector of residuals for the latent variables.

Using the SmartPLS 4.1 software (*41*), the SEM was developed. Five independent latent variables (road, horizontal curve, vertical curve, speed limit, and weather) and four moderator variables (age, education, income, and vehicle possession) were considered. The dependent variable is the crash severity outcome for the road segment, as defined earlier. The specification of paths in the SEM framework was based on established behavioral and traffic safety theories. Direct links between roadway and environmental characteristics, such as curvature, grade, speed limit, weather, and crash severity, are supported by geometric design and human factors principles. In addition, socioeconomic variables (income, education, age, and vehicle ownership) were included as both direct and moderating factors based on their known influence on driving behavior, exposure, and vehicle safety characteristics. For example, income can affect risk-taking tendencies and access to safer vehicles; education level is related to safety awareness and compliance with traffic laws; vehicle ownership affects exposure and mobility; and age distribution reflects differences in driving ability and risk perception. These logical relationships collectively support the structure of the paths shown in the following section, in Figure 1, ensuring that the model reflects the empirical evidence and established behavioral reasoning.

**Figure 1.**
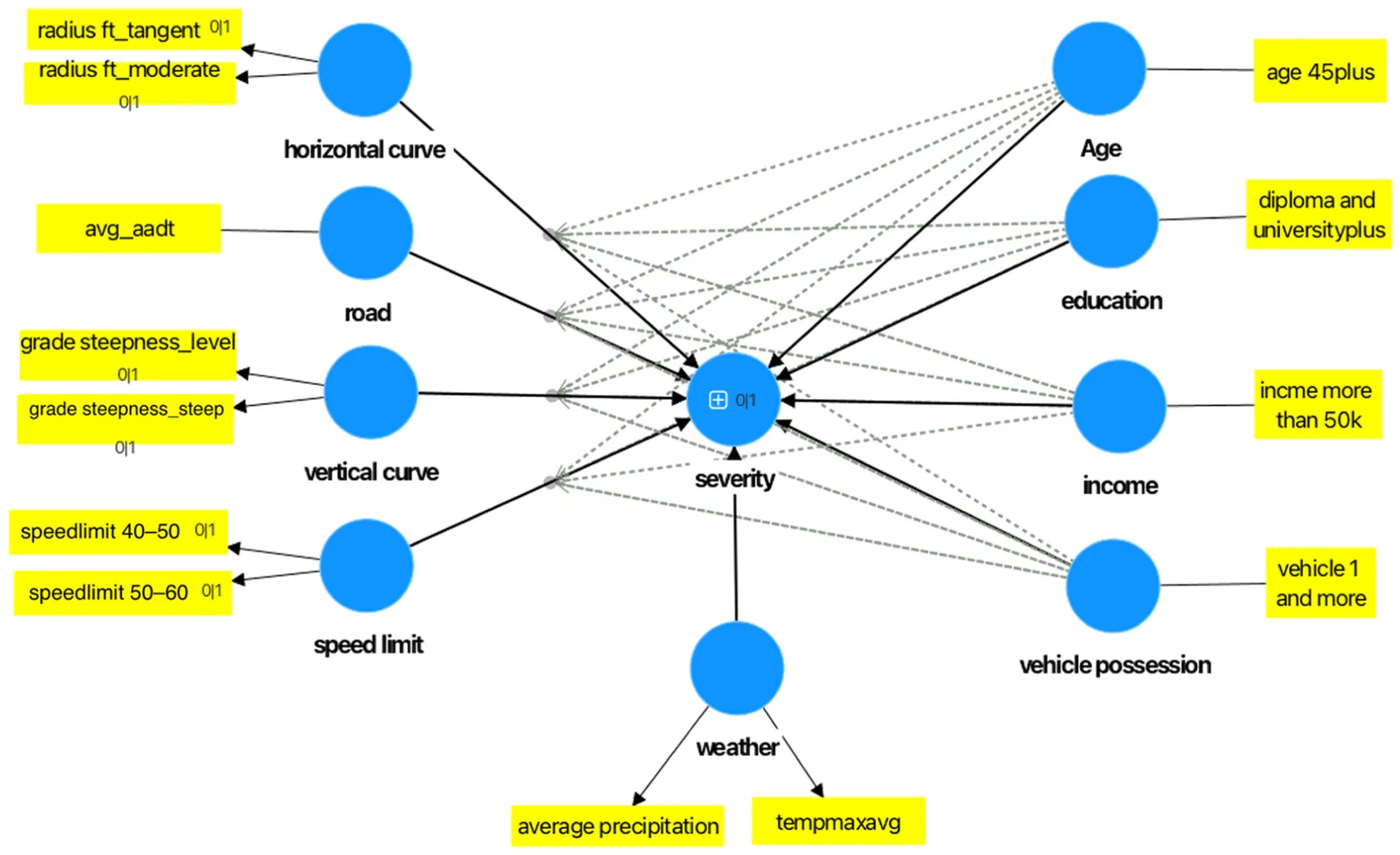
Structural Equation Modeling Model. *Note:* avg_aadt = annual average daily traffic; radius ft_tangent = curve radius (> 1,000 ft); radius ft_moderate = curve radius (500–1,000 ft); grade steepeness_level = grade percentage (0–3); grade steepness_steep = grade percentage (≥ 6); tempmaxavg = average annual maximum temperature (°F); speedlimit 40–50 = speed limit (mph); speedlimit 50–60 = speed limit (mph); age 45plus = proportion of population ages 45+; diploma and universityplus = proportion of population ages 25+ with a diploma and university degree; income more than 50k = proportion of households with income > £50,000; vehicle 1 and more = proportion of households with at least 1 vehicle.

In PLS–SEM, all constructs are modeled as latent, regardless of whether they are based on single or multiple indicators. Some variables initially included in the model would be excluded from the final structural model because of low reliability or validity metrics. To evaluate the quality of the SEM model, several criteria were assessed for each measurement model, including indicator reliability, composite reliability, convergent validity, and discriminant validity. The thresholds applied for these evaluations were based on the recommendations of Hair et al. (*20*). Indicator reliability was assessed by analyzing the loadings of indicators on latent variables with a standardized coefficient of at least 0.708. Consistency reliability was evaluated using the reliability coefficient $\rho_{a}$ , the minimum value of which should be between 0.6 and 0.7. Convergent validity was then assessed by calculating the average variance extracted (AVE), with a value of 0.5 or higher indicating that the indicators adequately explain the variance. Discriminant validity was evaluated using the Fornell–Larcker criterion, which compares the square root of AVE to the correlation coefficient between the latent variable and other variables. Finally, the structural models are evaluated using the coefficient of determination (*R*^2^). For more information on assessing PLS–SEM outcomes and the related criteria, refer to Hair et al. (*20*).

The PLS–SEM approach allows individual weights to be assigned to each attribute based on its importance and examines the hypothesis, reliability, and validity of measures. While SEM can capture linear relationships, it may oversimplify complex relationships. To address this limitation, ML techniques were used, which can model complex relationships to better understand the complexity. Therefore, PLS–SEM and ML are mathematically linked in a two-stage hybrid framework. In the first stage, PLS–SEM estimates the latent variable scores using its measurement and structural equations. In the second stage, these scores serve as the predictor input matrix for the ML models used to predict crash severity.

### Artificial Neural Network

A layered feed-forward back-propagation perception ANN using Python was employed to quantify the relationships between variables. In this approach, error minimization and improved estimation accuracy are achieved by calculating the discrepancy between actual and expected outcomes and feeding it back into the network to adjust synaptic weights. Input layer neurons correspond to the number of factors or inputs, while output layer neurons match the number of outputs or dependent variables (*12*, *14*, *43*). Similar to other ML methods in this study, the ANN received the latent variable scores extracted from the SEM as input, allowing the model to learn nonlinear patterns beyond the linear structure of the SEM framework. Further conceptual details on ANN and related ML techniques can be found in Bishop (*44*).

### Gradient Boosting with XGBoost

Gradient tree boosting with regularization is the algorithm that XGBoost uses (*45*). Gradient boosting is an ensemble learning algorithm that sequentially fits a simple base learner into the current model so that subsequent predictors can learn from the errors of previous predictors (*13*). It outperforms conventional tree-based models by improving the regularized objective and split-finding algorithms of this tree-based ensemble algorithm (*45*, *46, 47*). More information on this method can be found in Chen and Guestrin (*45*).

### Random Forest

RF is an ensemble learning method that generates multiple decision trees and aggregates their results (*48*). Decision trees are ML algorithms that utilize a tree-like structure to model data. It constructs multiple uncorrelated decision trees by randomly sampling features from the training data set. Each tree is built using a bootstrap sample of the data and a random subset of features. For predictions, categorical or classification problems rely on majority voting or the mode, while continuous or regression problems use the average of the predictions from all trees (*49*). In this study, the model was trained on latent variable scores from the SEM stage to capture nonlinear effects among predictors.

### Support Vector Machine

SVM, which was developed as an ML approach in 1992 by Boser et al. (*50*), is a binary classification algorithm that has been widely adapted to the recognition of patterns, including text and image recognition. The SVM algorithm determines an optimal segmentation hyperplane that separates two label sets of vectors, maximizing the distance between the hyperplane and the nearest vectors on either side (*22*). Data from two classes may not be linearly separable, in which case SVM can create a soft margin, a hyperplane separating many (but not all) data points (*51*). Similar to other techniques, the SVM in this study was trained using the latent variable scores from the SEM stage, enabling the detection of complex decision boundaries associated with crash severity outcomes.

## Results

The results are presented in three distinct, but complementary sections. The first section outlines the findings from the SEM analysis, the second compares the performance of different ML methods used in combination with SEM, and the last section ranks the factors affecting crash severity outcomes based on the best-performing model, which represents the main contribution of this study.

### SEM Results

Figure 1 shows the finalized SEM model as directly captured from the SmartPLS software. The independent latent variables consist of horizontal curvature, average annual daily traffic (AADT), speed limit ranges of 40–50 mph and 50–60 mph, vertical curvature, precipitation, and temperature. For the moderating factors, consideration was given to the demographic characteristics of the area where the crash happened, such as age, education level, household income exceeding $50,000, and vehicle ownership rates greater than one per household. The outcome variable, “severity,” is the crash severity outcome of a road segment, as defined earlier. Modeling these as latent variables in SEM allowed for better representation of the conceptual complexity of crash-influencing factors and moderators.

To initiate the SEM analysis, the reliability and validity of the constructs presented in Table 2 were evaluated. According to Hair et al., acceptable thresholds for composite reliability $\rho_{a}$ and AVE are 0.7 and 0.5, respectively (*20*). Furthermore, each indicator associated with a construct should have absolute loading values higher than 0.7. Based on these criteria, it can be confirmed from the results in Table 2 that all constructs exhibit satisfactory indicator reliability, internal consistency reliability, and convergent validity.

**Table 2.** Loadings, Reliability Coefficients, and Average Variance Extracted (AVE) Values of Constructs

Constructs	Indicators	Loading	Composite reliability (*ρ*_*a*_)	AVE
horizontal curve	radius ft_tangent radiusft_moderate	0.986 −0.786	1.452	0.796
spdlimt	speedlimit 40–50 speedlimit 50–60	−0.938 0.976	0.996	0.917
vertical curve	grade steepness_ level grade steepness_steep	0.977 −0.958	0.968	0.936
weather	average precipitation tempmaxavg	0.913 −0.866	0.760	0.792

*Note:* spdlimt = speed limit; radius ft_tangent = curve radius (> 1,000 ft); radiusft_moderate = curve radius (500–1,000 ft);grade steepeness_level = grade percentage (0–3); grade steepness_steep = grade percentage (≥ 6); speedlimit 40–50 = speed limit (mph); speedlimit 50–60 = speed limit (mph); tempmaxavg average = annual maximum temperature (°F).

To assess the discriminant validity of constructs, the Fornell–Larcker criterion was used. According to Table 3, the discriminant validity of latent variables is also achieved as the square root values of AVE (the diagonal values) are greater than the values of inter-construct correlation (the nondiagonal values).

**Table 3.** Discriminant Validity using Average Variance Extracted (AVE) Square Roots and Correlation Coefficients

Variable	Age	Education	Horizontal curve	Income	Road	Severity	Spdlimt	Vehicle possession	Vertical curve	Weather
Age	1	0.298	0.022	0.057	0.034	−0.002	−0.008	0.36	−0.008	0.032
Education	0.298	1	0.013	0.286	0.003	−0.017	0.067	0.501	0.05	0.003
Horizontal curve	0.022	0.013	0.892	0.089	0.136	0.026	0.021	0.023	0.115	0.141
Income	0.057	0.286	0.089	1	0.232	0	0.025	0.208	0.055	0.177
Road	0.034	0.003	0.136	0.232	1	0.047	−0.095	−0.025	0.03	0.228
Severity	−0.002	−0.017	0.026	0	0.047	1	0.041	0.007	0.059	0.045
Spdlimt	−0.008	0.067	0.021	0.025	−0.095	0.041	0.958	0.029	0.044	0.069
Vehicle possession	0.36	0.501	0.023	0.208	−0.025	0.007	0.029	1	0.053	−0.097
Vertical curve	−0.008	0.05	0.115	0.055	0.03	0.059	0.044	0.053	0.967	0.046
Weather	0.032	0.003	0.141	0.177	0.228	0.045	0.069	−0.097	0.046	0.89

*Note:* spdlimt = speed limit.

In the final step of the SEM model evaluation, *R*^2^, which is a measure of the proportion of variability explained by independent variables, was assessed. An *R*^2^ value of 0.012 was obtained, suggesting that the developed SEM model only explains a small portion of the variance in the data. Because *R*^2^ measures the strength of linear relationships, the low *R*^2^ values here suggest that linear components of the model explain only a limited proportion of the outcome variance. While this does not directly confirm the presence of nonlinear or complex relationships, it raises the possibility of nonlinear relationships that may exist and represent the need for further investigation using complementary modeling approaches. However, the use of PLS–SEM remains valuable in this context because of its ability to provide meaningful insights into the relationships between variables. Specifically, the PLS–SEM model facilitates analyzing latent variables, the extraction of latent scores, and the examination of indirect relationships and moderating effects. In summary, the results of the PLS–SEM analysis provide a foundation for further exploring the complexity of relationships using ML techniques.

Following confirmation of construct validity and reliability, the path coefficients can now be presented. These results, which are presented in Table 4 alongside their *p*-values, indicate that only a few of the relationships modeled in the SEM framework reveal significant associations between contextual and geometric factors. Of note, these relationships should be interpreted as associative rather than causal, given the observational nature of the data and absence of experimental controls.

**Table 4.** Path Coefficients of Structural Equation Modeling Model and Their Significance

Relationship	Coefficient	*p*-values
Age → severity	−0.006	0.639
Education → severity	−0.009	0.5
Horizontal curve → severity	0.012	0.315
Income → severity	−0.026	0.036
Road → severity	0.017	0.001
Spdlimt → severity	0.041	0.005
Vehicle possession → severity	0.014	0.294
Vertical curve → severity	0.047	< 0.001
Weather → severity	0.012	0.283
Age–vertical curve → severity	−0.004	0.713
Vehicle possession–vertical curve → severity	0.001	0.91
Education–road → severity	0.003	0.48
Income–spdlimt → severity	−0.002	0.9
Income–horizontal curve → severity	0.017	0.287
Education–horizontal curve → severity	0.001	0.927
Income–vertical curve → severity	0.029	0.008
Age–spdlimt → severity	−0.005	0.701
Education–spdlimt → severity	−0.012	0.387
Vehicle possession–spdlimt → severity	0.009	0.514
Vehicle possession–horizontal curve → severity	−0.014	0.361
Vehicle possession–road → severity	0.001	0.89
Income–road → severity	−0.001	0.801
Education–vertical curve → severity	0.009	0.373
Age–horizontal curve → severity	0.012	0.408
Age–road → severity	0.001	0.876

*Note:* spdlimt = speed limit.

The SEM results suggest that most interactions and moderation effects are not significant. This outcome may be attributed to weak associations and to the underlying nonlinear and complex relationships that SEM’s linear modeling framework is not designed to capture. In particular, crash severity outcomes are influenced by intricate interrelationships between roadway characteristics, environmental conditions, and neighborhood characteristics, which may involve nonadditive combinations. These forms of nonlinearity can emerge from the nature of the data (e.g., skewed distributions or contextual interactions) or from structural limitations of the model (e.g., assuming linearity where it does not hold). The ML models are better suited for uncovering such nonlinear patterns because they do not require a priori specification of functional forms and can flexibly model complex, high-dimensional interactions.

### Model Performance Analysis

As noted, ML methods were applied to complement the SEM findings in exploring whether such a data-driven approach could reveal stronger relationships not captured in the linear SEM framework. The ML models extend the SEM analysis by capturing nonlinear moderation effects that may not appear significant within the linear SEM framework. By learning complex dependencies among the latent variables, ML provides complementary evidence on how socioeconomic factors influence the relationships between road and weather conditions and crash severity. This enables the combined SEM–ML approach to evaluate linear and nonlinear moderation patterns within a unified framework. Recognizing that the performance of different methods may vary when used in conjunction with SEM to analyze the moderation effects, the different techniques were compared to select the best one. The data were randomly split into training and testing data sets with a 70:30 ratio to analyze the crash severity. Training data sets were used to train the models, which were then applied to the testing data set to predict crash severity outcomes for road segments.

Hyperparameter tuning, which is the process of optimizing the hyperparameters of an ML model to improve its performance, was performed as one of the important steps in developing ML methods. These hyperparameters are configuration settings defined before training a model and remain fixed during the training process. Bayesian optimization (BO), a powerful technique for hyperparameter tuning in ML models, was employed for this to determine the most efficient model structures to prevent over- and under-fitting. BO is particularly useful when dealing with expensive or time-consuming optimization problems where evaluating the objective function is computationally demanding. The hyperparameter values of the model parameters after BO are given in Table 5.

**Table 5.** Hyperparameter Values of the Models Using Bayesian Optimization

Parameters	Description of parameters	Values
ANN		
activation	Nonlinear activation function	elu
batch_size	Number of training samples processed before updating weights	335
dropout	Dropout regularization	0.436
dropout_rate	Fraction of neurons randomly dropped during training	0.23
epochs	Number of complete passes through the training dataset	44
layers1	Number of neurons in the first hidden layer	1
layers2	Number of neurons in the second hidden layer	1
learning_rate	Step size to update model weights	0.426
neurons	Total number of neurons per layer	31
normalization	Whether input data are normalized before training	Yes
RF		
n_estimators	Number of trees in the forest	159
max_depth	Maximum depth allowed for each decision tree	32
min_samples_split	Minimum number of samples required to split a node	2
max_features	Fraction of features considered when splitting a node	0.1
XGBoost		
colsample_bytree	Fraction of predictor variables sampled for each tree	0.852
gamma	Minimum loss reduction	2.62
max_depth	Maximum depth of each tree	15
min_child_weight	Minimum sum of weights needed in a child node	9
reg_alpha	L1 regularization term on leaf weights	123
reg_lambda	L2 regularization term on leaf weights	0.74
SVM		
C	Regularization parameter controlling trade-off between margin width and misclassification error	2.81
Gamma	Parameter defining the influence of a single training example	0.98

*Note:* ANN = artificial neural network; RF = random forest; XGBoost = eXtreme gradient boosting; SVM = support vector machine.

After identifying the optimal structure of ML techniques, the performance metrics of different models were identified. Because the crash data are inherently imbalanced, precision, recall, and F1-score are used as the performance measures (*52*). In addition, when performing multiclass classification, the overall effectiveness of these metrics across all classes must be evaluated, so the macro average (Macro AVG) is typically utilized (*52*).

Table 6 summarizes the performance measures of different data mining algorithms. Of note, the dependent variable exhibited moderate imbalance (15% severe crashes); therefore, hyperparameter tuning was conducted with class-sensitive settings, and model performance was assessed using metrics that are robust to imbalance (macro-averaged precision, recall, and F1-score). Because the objective of this study was to evaluate the methodological framework rather than to maximize predictive accuracy, no additional resampling was applied. However, future research focused on prediction-oriented optimization could incorporate approaches such as the synthetic minority oversampling technique (SMOTE) (*53*), a resampling technique that enhances sensitivity to minority-class events by creating synthetic, unique samples based on the existing ones.

**Table 6.** Accuracy of Applied Data Mining Techniques

Performance metrics	Macro AVG	Weighted AVG
SEM–ANN		
Precision	0.43	0.73
Recall	0.5	0.85
F1-score	0.46	0.79
SEM–XGBoost		
Precision	0.43	0.73
Recall	0.5	0.85
F1-score	0.46	0.79
SEM–SVM		
Precision	0.54	0.77
Recall	0.51	0.82
F1-score	0.51	0.77
SEM–RF		
Precision	0.55	0.77
Recall	0.52	0.83
F1-score	0.52	0.79

*Note:* ANN = artificial neural network; RF = random forest; XGBoost = eXtreme gradient boosting; SVM = support vector machine; AVG = average; SEM = sequential equation modeling.

Table 6 highlights that, compared with other data mining algorithms, RF performed relatively well when complemented with SEM, followed by SVM, and that ANN and XGBoost have the lowest accuracy when combined with SEM. While RF achieved the best overall performance among the tested models, the relatively strong performance of SVM offers useful insight into the nature of the relationships in the data. This is because SVM is particularly effective in handling high-dimensional, nonlinear classification problems and is known for its robustness in small-to-moderate sample sizes with noisy data. Its strong performance suggests that the decision boundaries separating different crash severity outcomes may be nonlinear but relatively smooth and separable using kernel-based methods. In contrast, the slightly weaker performance of ANN may be because of its sensitivity to training parameters and potential overfitting in data sets with class imbalance and complex variable interactions. XGBoost, while powerful in capturing gradient-based decision structures, may have been less effective in this case because of the presence of mixed data types and potential noise in some socioeconomic indicators. These differences in performance underscore the value of testing multiple models because each offers distinct capabilities in capturing relationships within different portions of the feature space. Of note, these findings are specific to the characteristics of the current data set, and investigations using other data sets, potentially with different distributions and variable types, may lead to different conclusions on the relative performance of these algorithms.

Of note, the PLS–SEM and ML models evaluate performance using different criteria. The *R*^2^ values from the SEM stage represent the proportion of variance explained by linear structural relationships, whereas the data-driven performance metrics reported for the ML models reflect predictive performance for classification using latent variable scores as inputs. Because these measures are derived from different modeling frameworks, they are not directly comparable. However, the ML results demonstrate improved predictive ability and broader representation of nonlinear moderation effects beyond the linear SEM structure.

### Feature Importance Analysis

Given its superior performance compared with other data mining algorithms, RF was utilized to rank the variables based on their effect on crash severity. To better interpret the results from feature importance using RF, the SHAP algorithm was utilized. The SHAP algorithm (*15*), a game theory-based approach, addresses the need for interpretability by quantifying the effect of each feature on predictions (*54*). By assigning a specific value to each feature, SHAP quantifies the effect of that feature on the final prediction, offering a comprehensive understanding of the model’s decision-making process (*22*).

Figure 2 shows the most important predictors of crash severity outcomes on road segments based on the SEM–RF algorithm. The graph presented in this figure offers a visual representation of the importance of features using SHAP values. The vertical axis represents the different features, and the horizontal axis displays their corresponding SHAP values, indicating the degree of influence each feature has on the model predictions. The SHAP value for each feature is associated with a specific color, as shown in the color bar on the right. Red dots signify higher crash risk factors, whereas blue dots indicate lower crash risk factors, providing a clear visualization of the effect of each feature on the prediction results (*22*, *54*).

**Figure 2.**
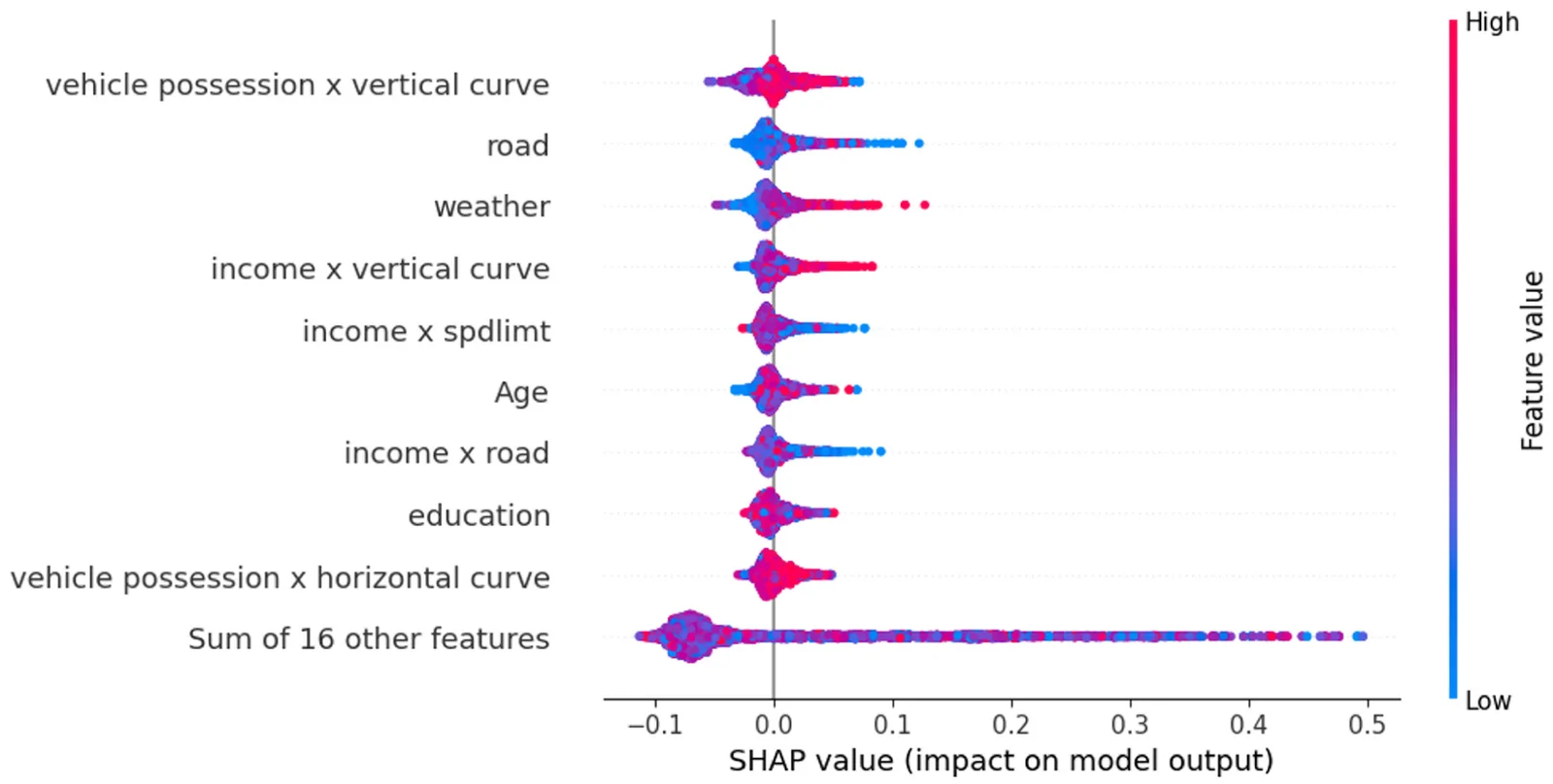
Feature importance derived from Shapley additive explanation algorithm (SHAP). *Note:* spdlimt = speed limit.The moderation effects are shown using “×”.

As shown in Figure 2, the “vehicle possession × vertical curve” appears to be the most effective feature, suggesting that a higher percentage of vehicle possession in the neighborhood interacts significantly with road vertical grades in affecting crash severity. This shows that segments located in high vehicle ownership and higher vertical grades are associated with higher predicted crash outcomes. This effect may be because of several underlying factors, such as driver behavior. “Road” and “weather” are also highly influential, indicating that external road and environmental conditions play a crucial role in crash severity. For weather, the positive SHAP values for higher “weather” conditions indicate an increased likelihood of severe crashes. Of interest, a literature review by Theofilatos and Yannis (*55*) revealed that precipitation does not appear to have a consistent effect on the severity and that the effect of other weather parameters on safety was not found to be straightforward. In the case of the “road” variable, the presence of both red (high feature value) and blue (low feature value) dots on the right side of the SHAP plot suggests that the relationship between “road” (e.g., AADT) and crash severity is nonlinear. This is consistent with the findings in the literature, for example, Liang et al. (*56*), who found that the effect of AADT on crash severity is not linear and varies depending on roadway and traffic conditions. The other important factor is “income × vertical curve,” which shows a similar trend, indicating that segments located in high-income neighborhoods and sharper vertical curvature are associated with higher predicted crash outcomes. This is possibly because higher-income drivers might be driving at higher speeds. Finally, from the results shown in Figure 2, some of the moderation effects (e.g., vehicle possession × vertical curve, vehicle possession-horizontal curve, income × road, and income × speed limit) are among the most important parameters in the model, which were not captured in the SEM model.

## Summary and Conclusions

The objective of this study was to explore a novel hybrid PLS–SEM and ML technique for predicting traffic crash severity. Unlike traditional statistical models that struggle with complex, nonlinear relationships, this hybrid approach integrates the explanatory power of SEM with the predictive accuracy of ML methods.

For this study, a database of Ohio collector road segments was used as an illustrative case study to assess the moderation effects of socioeconomic factors in predicting severe crash outcomes based on road and environmental characteristics. The exploration of these moderation effects adds to the novelty of this study.

Central to this study was a comparative evaluation of the predictive performance of hybrid two-stage models, where SEM captured moderating relationships and ML techniques were used to analyze crash severity. A subsequent feature importance analysis using the SHAP algorithm revealed nuanced interactions, such as those between socioeconomic factors and road characteristics, that traditional models may overlook.

The case study is novel in that it comprehensively explores the interplay between road, weather, and socioeconomic factors in traffic crash analysis using a two-stage modeling framework. From a policy perspective, the illustrative insights show how agencies could prioritize interventions at locations where geometric and socioeconomic characteristics intersect. For instance, the interplay between income and speed limit points to the need for equitable roadway design and speed management strategies in socioeconomically diverse neighborhoods. These insights can inform systemic safety interventions by helping agencies prioritize investments in locations where geometric and socioeconomic vulnerabilities intersect. Furthermore, data-driven tools, such as the hybrid framework proposed in this study, could be incorporated into the optimization of network screening and Vision Zero initiatives to ensure that limited safety resources are deployed equitably and effectively.

## Limitations and Future Studies

This study has limitations from the application perspective. First, the case study application did not incorporate explicit driver-specific and behavioral factors, which can be critical determinants of crash severity. Although certain socioeconomic and environmental indicators may serve as partial proxies for these behavioral factors, the absence of direct measures constrains the ability to fully capture the influence of human factors on crash outcomes. Integrating real time driving behavior data, such as connected vehicle records, eye-tracking metrics, and driver reaction measures, would further enhance predictive accuracy and provide a more complete understanding of the mechanisms influencing crash severity. Second, the analysis was limited to Ohio collector road segments, which restricts the generalizability of the findings to other geographic areas and roadway classifications. Variations in road design, traffic patterns, regulatory environments, and socioeconomic contexts in other settings may produce different results. Future research should address this limitation by expanding the geographic and contextual scope to include diverse roadway classifications, collision types, and regions, improving external validity. In addition, the application revealed a potential issue with the current modeling framework in the imbalance in the crash severity outcome. Although the ML models were configured with class weighting and other imbalance-sensitive parameters, and performance was evaluated using class-sensitive metrics, it may still be of interest for future studies to explore the need for resampling techniques, such as SMOTE, for addressing data imbalance. Finally, crash severity was treated as a binary outcome by distinguishing segments with at least one injury or fatal crash from those with property damage only crashes. This definition aligns with the systemic safety approach and simplifies interpretation; it does not capture certain details, such as the frequency of different injury levels. Future studies could explore the consequences of this simplification by applying measures, such as Equivalent Property Damage Only crash frequency or other severity-weighted indices. There could be value in such an approach in that it would preserve more information about severity variation across segments while maintaining compatibility with systemic safety screening and prioritization frameworks.
